# The incidences of metachronous multiple gastric cancer after various types of gastrectomy: analysis of data from a nationwide Japanese survey

**DOI:** 10.1007/s10120-020-01104-1

**Published:** 2020-07-12

**Authors:** Shinichi Kinami, Masaki Aizawa, Hiroharu Yamashita, Koshi Kumagai, Satoshi Kamiya, Makoto Toda, Takaomi Takahata, Muneharu Fujisaki, Hiroshi Miyamoto, Hiroshi Kusanagi, Kenta Kobayashi, Marie Washio, Kei Hosoda, Takeo Kosaka

**Affiliations:** 1 Department of Surgical Oncology, Kanazawa Medical University, 1-1 Daigaku, Uchinada-machi, Kahoku-gun, Ishikawa, 920-0293 Japan; 2 Department of Digestive Surgery, Niigata Cancer Center Hospital, Niigata, Japan; 3 Department of Gastrointestinal Surgery, The University of Tokyo, Tokyo, Japan; 4 Department of Gastroenterological Surgery, Cancer Institute Hospital, Japanese Foundation for Cancer Research, Tokyo, Japan; 5 Department of Gastric Surgery, Shizuoka Cancer Center, Shizuoka, Japan; 6 Department of Surgery, Yamagata Prefectural Central Hospital, Yamagata, Japan; 7 Department of Surgery, Okayama Saiseikai General Hospital, Okayama, Japan; 8 Department of Surgery, Jikei University School of Medicine, Tokyo, Japan; 9 Department of Surgery, Gastroenterological Center, Yokohama City University Medical Center, Kanagawa, Japan; 10 Department of General Surgery, Kameda Medical Center, Chiba, Japan; 11 Department of Gastric Surgery, Tokyo Medical and Dental University Hospital, Tokyo, Japan; 12 Department of Upper Gastrointestinal Surgery, Kitasato University, Kanagawa, Japan

**Keywords:** Metachronous gastric cancer, Proximal gastrectomy, Pylorus preserving gastrectomy, Function preserving gastrectomy, Endoscopic submucosal dissection

## Abstract

**Background:**

The incidence of metachronous multiple gastric cancer (MMGC) after gastrectomy remains unclear. This study evaluated the incidences of MMGC according to specific gastrectomy types, including pylorus-preserving gastrectomy (PPG), proximal gastrectomy (PG), and function-preserving gastrectomy (FPG), which was categorized as segmental gastrectomy and local resection.

**Methods:**

We conducted a questionnaire survey of the Japanese Society for Gastro-Surgical Pathophysiology members, who were asked to report their institutional numbers of radical gastrectomy cases for cancer between 2003 and 2012. The cases were categorized according to whether the remnant stomach’s status was followed for > 5 years, confirmation of MMGC, time to diagnosis, and treatment for MMGC. We calculated the “precise incidence” of MMGC by dividing the number of MMGC cases by the number of cases in which the status of remnant stomach was followed up for > 5 years.

**Results:**

The responses identified 33,731 cases of gastrectomy. The precise incidences of MMGC were 2.35% after distal gastrectomy (DG), 3.01% after PPG, 6.28% after PG (*p* < 0.001), and 8.21% after FPG (*p* < 0.001). A substantial proportion of MMGCs (36.4%) was found at 5 years after the initial surgery. The rates of MMGC treatment using endoscopic submucosal dissection were 31% after DG, 28.6% after PPG, 50.8% after PG (*p* < 0.001), and 67.9% after FPG (*p* < 0.001).

**Conclusions:**

The incidence of MMGC was 2.4% after DG, and higher incidences were observed for larger stomach remnants. However, the proportion of cases in which MMGC could be treated using endoscopic submucosal dissection was significantly higher after PG and FPG than after DG.

## Introduction

Adenocarcinomas often develop in the remnant stomach after gastrectomy [1–4], which are collectively referred to as “cancer in the remnant stomach” [4]. Historically, most cases involved gastric stump carcinoma after surgery for peptic ulcers [1–4], although more recent cases tend to involve metachronous multiple adenocarcinoma after gastrectomy for gastric cancer [2–7]. The incidence of metachronous multiple gastric cancer (MMGC) may also be higher than that of primary gastric cancer [5]. This is because the gastric mucosa of patients with a history of gastric cancer is thought to have accumulated carcinogenic factors, such as genetic predisposition and damage caused by the gastric environment, relative to the gastric mucosa of people with no history of gastric cancer [6]. Thus, it is important to accurately diagnose and treat MMGC. As most recurrences of the initial cancer occur within 5 years after curative resection, there is a limited amount of clear follow-up data from > 5 years after the initial gastrectomy. Therefore, the incidence of MMGC, time from initial gastrectomy to MMGC diagnosis, and related risk factors remain unclear [7–9].

The standard gastrectomy procedures are distal partial gastrectomy (DG) and total gastrectomy (TG), although post-gastrectomy syndromes are important issues that are related to these procedures [10–13]. In East Asia, limited surgeries (modified surgeries) are common based on the recommendations of the Japanese guideline [14], such as pylorus-preserving gastrectomy (PPG) and proximal gastrectomy (PG). Furthermore, function-preserving gastrectomy (FPG) with a minimal resection area, such as segmental gastrectomy (SG) and local resection (LR), has been attempted [15, 16]. However, the relationship between the size of the remaining gastric mucosa and the incidence of MMGC is unclear [6]. The risk of MMGC would presumably be greater with a larger amount of remaining gastric mucosa, which would suggest that the incidence of MMGC would be higher after these limited surgeries and FPG, relative to standard gastrectomy [6]. Nevertheless, few studies have evaluated this topic. Therefore, we conducted a questionnaire survey of Japanese centers that specialize in treating gastric cancer and analyzed prospectively collected data from a large sample of cases involving gastrectomy for gastric cancer to evaluate the occurrence of MMGC. We also aimed to estimate the precise incidences of MMGC in relation to the specific gastrectomy procedures, especially for PPG, PG, and FPG.

## Methods

The Japanese Society for Gastro-Surgical Pathophysiology (JSGSP) was founded in 1972 as a research society for vagotomy and is a membership-based academic group that is dedicated to the study of gastric surgery and post-gastrectomy disorders (https://www.jsgp.jp/index.php). Institutions that participate in the JSGSP must have a full-time physician specializing in gastric surgery. In accordance with the presidency of the JSGSP 48th Annual Meeting, a nationwide questionnaire survey was planned by the JSGSP president (TK) and this study was supervised by SK. The questionnaire only considered the number of cases for each questionnaire item and did not collect any identifiable patient information. The JSGSP members accessed the web-based questionnaire between May 2018 and October 2018, and returned the responses via e-mail. The raw data were checked by SK for input errors and responses with no errors were considered valid and sent to Convention Linkage, Inc. (Tokyo Japan) for compilation.

The study protocol was approved by the ethics committee of Kanazawa Medical University (Trial No. I267) and complied with the Good Clinical Practice guidelines and the Declaration of Helsinki. All data were anonymized and compiled for each facility, although data aggregation was subject to the judgment and approval of each facility's ethics committee. Patients were allowed to opt out of the research use of their data at any time.

**Fig. 1:**
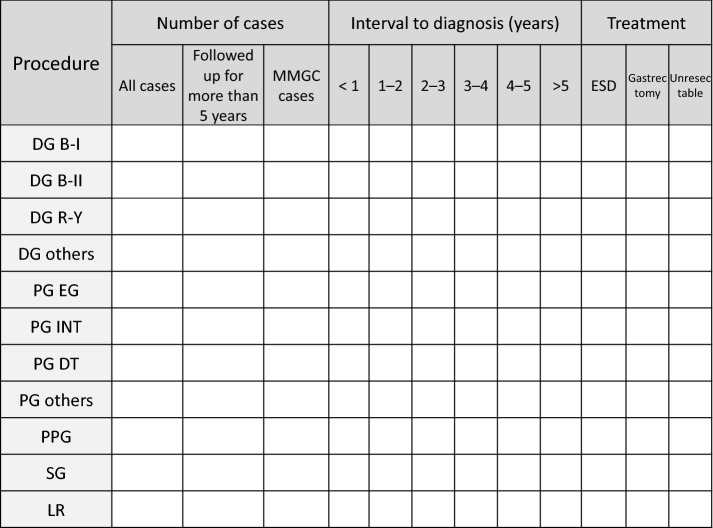
The questionnaire that was used in this article (English translation).We would like to ask about the occurrence of metachronous multiple gastric cancer after radical surgery for gastric cancer in your hospital during 2003–2012 (please only note recorded cases). Fill in the blanks in the table below with the number of cases. “Followed more than 5 years” describes patients whose MMGC status (yes/no) was confirmed at > 5 years using routine endoscopy. This includes cases in which the gastric cancer recurred and the patient died but does not include patients who were lost to follow-up within 5 years or patients who survived but did not undergo endoscopy. *MMGC* metachronous multiple gastric cancer, *ESD* endoscopic submucosal dissection, *DG* distal gastrectomy, *B-I* Billroth I, *B-II* Billroth II, *R-Y* Roux-en Y, *PG* proximal gastrectomy, *EG* esophago-gastric anastomosis, *INT* jejunal interposition, *DT* double tract reconstruction, *PPG* pylorus-preserving gastrectomy, *SG* segmental gastrectomy, *LR* local resection of stomach

The questionnaire was divided into three sections, and this report summarizes the data collected in the second section. Figure 1 shows an English translation of the questionnaire sheet. Participating institutions were asked to indicate the number of patients who underwent radical resection for initial gastric cancer between 2003 and 2012, as well as the numbers of cases involving the different surgical procedures. The questionnaire also asked the institutions to indicate the number of cases in which the remnant stomach’s status was followed for > 5 years, the number of confirmed MMGC cases, the time from the gastrectomy to the diagnosis of MMGC, and the treatments for MMGC. In this study, “cases in which the remnant stomach’s status was followed up for > 5 years” were used to identify patients whose MMGC status (yes/no) was judged based on the remnant stomach’s status at > 5 years using routine endoscopy. This included patients who experienced gastric cancer recurrence and subsequently died within 5 years but did not include cases without 5 years of follow-up or cases in which survival was confirmed but endoscopy was not performed. The resection and reconstruction methods were considered for the surgical procedures. Tumors diagnosed as adenocarcinoma by endoscopic biopsy and not as recurrence of the initial cancer were defined as MMGC. Only the first diagnosed MMGC was considered.

The analyses involved two methods for calculating the incidence of MMGC. The “crude incidence” was defined as the total number of MMGCs divided by the total number of gastrectomy cases. To more accurately calculate the incidence, we also calculated the "precise incidence" as the number of MMGC cases divided by the number of cases in which the remnant stomach’s status was followed up for > 5 years. In this calculation, we excluded cases without full endoscopic follow-up, such as those in which endoscopic examination was not possible or those that were lost to follow-up.

Inter-group comparisons were performed using the chi-squared test, and differences were considered statistically significant at *p* values of < 0.05. All statistical analyses were performed using EZR (Saitama Medical Center, Jichi Medical University, Saitama, Japan), which is a graphical user interface for R software (The R Foundation for Statistical Computing, Vienna, Austria) that involves a modified version of R Commander that includes frequently used biostatistical functions [17].

## Results

**Fig. 2:**
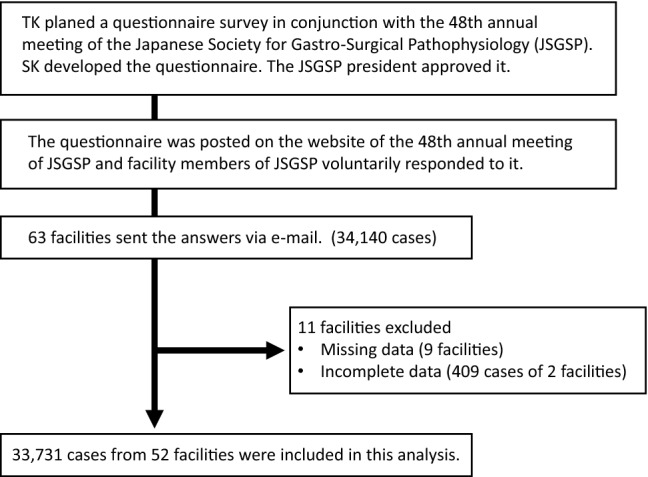
The flow diagram of the investigation

**Fig. 3:**
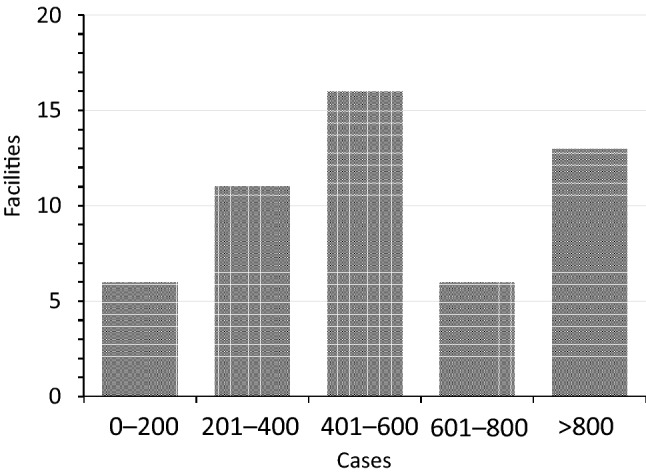
The numbers of initial gastrectomy cases that were reported by each facility. The most common number of cases was 400–600 cases per facility, which was followed by > 800 cases per facility

We received questionnaire responses from 63 facilities, although responses from 11 facilities were excluded because of missing or inconsistent data (probably these facilities made a simple calculation error). Thus, data were compiled from 52 facilities, which are indicated in the Acknowledgements section with the names of reporting individuals. Figure 2 is a flow chart of this study. Figure 3 shows the numbers of surgeries reported by each facility, with a median number of 504 cases per institution and a maximum number of 2410 cases per institution.

A total of 33,731 cases were reported, including 24,451 cases in which the remnant stomach’s status was followed up for > 5 years. The crude incidence of MMGC was 2.12% (718 cases) and the precise incidence was 2.94%. Table 1 summarizes the incidences of MMGC according surgical procedure. In the questionnaire, SG and LR were considered separately but were grouped together as FPG for this analysis. The statistical tests considered DG as the reference procedure and revealed that the incidence of MMGC varied according to the different procedures. The precise incidences of MMGC were 2.35% after DG, 6.28% after PG (2.7-fold higher), and 8.21% after FPG (3.5-fold higher). The precise incidence was 3.01% after PPG (1.3-fold higher), although this increase was not statistically significant. The proportions of cases with > 5-year follow-up of the remnant stomach’s status were significantly higher among PPG and FPG cases than among DG and PG cases.

**Table 1:** Incidences of metachronous multiple gastric cancer according to surgical procedure

Procedure	All cases	Cases with 5-year follow-up	% followed (%)	MMGC cases	% of MMGC	
					Crude (%)	Precise (%)
DG	27,298	19,530	71.5	459	1.68	2.35
PPG	2644	2093	79.2**	63	2.38*	3.01
PG	2603	1878	72.1	118	4.53**	6.28**
FPG	1186	950	80.1**	78	6.58**	8.21**

Statistical tests used the DG group as the reference. *p < 0.05; **p < 0.001

Cases with 5-year follow-up were defined as cases in which the MMGC status (yes/no) was confirmed at > 5 years using routine endoscopy. The precise incidence of MMGC was defined as the number of MMGC cases divided by the number of cases with remnant stomach status that was followed for > 5 years

*MMGC* metachronous multiple gastric cancer, *DG* distal gastrectomy, *PPG* pylorus-preserving gastrectomy, *PG* proximal gastrectomy, *FPG* function-preserving gastrectomy

Table 2 shows the precise incidences according to the reconstruction method after DG and PG (reconstruction methods with < 100 cases were omitted). The incidences of MMGC were 2.72% after Billroth I (B-I) and 3.17% after Billroth II (B-II), which were not significantly different, although a significantly lower incidence (1.55%) was observed after Roux-en Y (R-Y). The incidence after R-Y was also significantly lower than after B-II. However, there were no significant differences in the incidences of MMGC among the reconstruction methods after PG. Moreover, there was no significant difference in the incidence of MMGC between SG and LR.

**Table 2:** The precise incidences of metachronous multiple gastric cancer according to specific type of distal gastrectomy, proximal gastrectomy, and function-preserving gastrectomy

Procedure	Cases with 5-year follow-up	MMGC cases	% of MMGC
DG			
B-I	12,214	332	2.72
B-II	947	30	3.17
R-Y	6271	97	1.55*
PG			
EG	896	65	7.25
INT	527	31	5.88
DT	399	17	4.26
FPG			
SG	189	14	7.41
LR	761	64	8.41

Reconstruction methods with < 100 cases were omitted

*MMGC* metachronous multiple gastric cancer, *DG* distal gastrectomy, *B-I* Billroth I, *B-II* Billroth II, *R-Y* Roux-en Y, *PG* proximal gastrectomy, *EG* esophago-gastric anastomosis, *INT* jejunal interposition, *DT* double tract reconstruction, *FPG* function-preserving gastrectomy, *SG* segmental gastrectomy, *LR* local resection of stomach

*The incidence of R-Y was significantly less than that of B-I (*p* < 0.001) and B-II (*p* < 0.01)

**Fig. 4:**
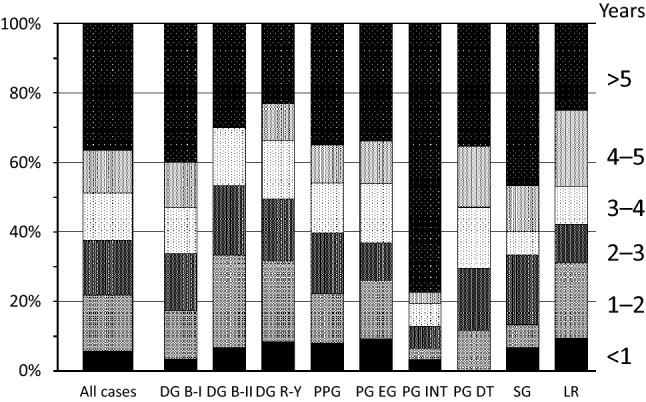
The intervals from the initial gastrectomy to the diagnosis of metachronous multiple gastric cancer. The leftmost bar shows the ratios for all cases, and the other bars show the ratios for each surgical procedure. *DG* distal gastrectomy, *B-I* Billroth I, *B-II* Billroth II, *R-Y* Roux-en- Y, *PPG* pylorus-preserving gastrectomy, *PG* proximal gastrectomy, *EG* esophago-gastric anastomosis, *INT* jejunal interposition, *DT* double tract reconstruction, *SG* segmental gastrectomy, *LR* local resection of stomach

Figure 4 shows the times from the initial gastrectomy to the MMGC diagnosis, which indicated that 36.4% of the MMGCs were found at > 5 years after the initial surgery. The patterns of the intervals from the initial gastrectomy to the diagnosis of MMGC were similar for most surgical procedures, although the interval after PG with jejunal interposition reconstruction (INT) was significantly different from that after DG B-I (*p* = 0.007).

Table 3 shows the treatments for MMGC, which were classified as endoscopic submucosal dissection (ESD), gastrectomy, or none (unresectable). The analyses for DG were performed according to the reconstruction method because of the low incidence of MMGC after R-Y, although PG and FPG were evaluated together because there was no significant difference in the incidences of MMGC between the reconstruction methods or procedures. Among the DG cases, there was no significant difference in treatment for MMGC between the reconstruction methods. The rate of ESD for MMGC were 50.8% after PG and 67.9% after FPG, and the rates of re-gastrectomy were significantly lower than those after DG. The rates of ESD were similar after PPG and after DG B-I, although the absence of unresectable cases was responsible for the significant difference.

**Table 3:** The treatments for metachronous multiple gastric cancer

Procedure	Treatment			% of DdMMGC
	ESD (%)	Gastrectomy (%)	Unresectable (%)	
DG B-I	31.0	60.2	8.7	1.87
DG B-II	20.0	63.3	16.7	2.53
DG R-Y	39.2	50.5	10.3	0.94^c^
PPG	28.6^a^	71.4	0	2.15
PG	50.8^c^	42.4	6.8	3.09^b^
FPG	67.9^c^	32.1	0	2.63

Statistical tests used the DG B-I group as the reference within each treatment comparison

*DdMMGC* disadvantaged cases due to metachronous multiple gastric cancer, which included unresectable cases or cases requiring re-gastrectomy, *DG* distal gastrectomy, *B-I* Billroth I, *B-II* Billroth II, *R-Y* Roux-en Y, *PPG* pylorus-preserving gastrectomy, *PG* proximal gastrectomy, *FPG* function-preserving gastrectomy

^a^*p* < 0.05; ^b^*p* < 0.01; ^c^*p* < 0.001

We defined cases that required re-gastrectomy plus unresectable cases as disadvantaged cases due to MMGC (DdMMGC). The incidence of DdMMGC was calculated based on the number of cases in which the remnant stomach’s status was followed for > 5 years (the denominator). The incidence of DdMMGC after DG B-I was 1.87%, which was not significantly different than that after B-II (2.53%) but was significantly higher than that after R-Y (0.94%). The incidence of DdMMGC after PG was 3.09%, which was significantly higher than that after DG B-I. There were no significant differences in the incidences of DdMMGC after PPG, FPG, and DG B-I.

## Discussion

The generic term for cancer arising in the remnant stomach after gastrectomy is “cancer in the remnant stomach”, which can be grouped into various types. For example, cancer arising from the remnant stomach after gastrectomy for benign disease is referred to as gastric stump carcinoma. There has been interest in determining whether the mechanism of carcinogenesis is different between gastric stump carcinoma and whether the prognosis of gastric stump carcinoma is poorer than that of normal gastric cancer located in the upper-third of the stomach [1–4]. However, many patients who underwent gastrectomy for peptic ulcers have subsequently died, and most cases of “cancer in the remnant stomach” are now considered MMGC.

The incidence of MMGC, time from initial gastrectomy to MMGC diagnosis, and related risk factors have been unclear [5–8]. The incidence of MMGC after DG was estimated to be approximately 2% in Japan 20 years ago [5], although an accurate assessment requires prospective collection of a large sample of cases with complete endoscopic follow-up. Hanyu et al. performed a long-term follow-up of 437 cases involving DG B-I and used the Kaplan–Meier curves to estimate the cumulative MMGC incidences, which revealed incidences of 3.7% at 10 years and 5.4% at 20 years [18]. Iwata et al. followed up 511 DG cases and 93 PG cases and reported 5-year cumulative MMGC incidences of 2.3% after DG and 6.8% after PG [19]. However, these results were limited by the numbers of cases that could be accumulated at a single center, and we are not aware of any reports regarding the incidence of MMGC after SG or LR.

To address this issue, we planned a questionnaire survey in conjunction with the 48th annual JSGSP meeting, which ultimately accumulated 33,731 cases. The inclusion criteria for this study were all gastric cancer surgery cases performed in each participating institute from 2003 until 2012. There were no exclusion criteria. There are two reasons for conducting a questionnaire to the members of JSGSP: (1) it provides reliable data and (2) it allows us to collect data evenly from all over Japan. These data can be interpreted as standard data of the Japanese patients. The survey also provided good samples of cases that involved limited surgeries and FPG, such as PG (2603 cases), PPG (2644 cases), SG (189 cases), and LR (761 cases). We are not aware of any other studies that have evaluated MMGC based on such a large sample, and data accuracy is likely high because the survey included many high-volume centers with a high degree of expertise and careful follow-up. Nevertheless, it is difficult to calculate the cumulative incidence of MMGC in such large-scale studies as the incidence increases over time, and incidence estimate curves must be considered [8, 9, 18, 19]. Rather than the cumulative incidence, this study evaluated the “precise incidence” by dividing the total number of MMGC cases by the number of cases with > 5-year follow-up of the remnant stomach’s status. The difference between “crude incidence” and “precise incidence” was whether or not the case deviated from the follow-up program. The latter is a case in which annual endoscopy was performed for more than 5 years in accordance with the Japanese guidelines for gastric cancer treatment and the regional cancer treatment clinical path. The “precise incidence” of MMGC was 2.35% after DG, which should be higher than the 5-year cumulative incidence rate and lower than the 15-year cumulative incidence rate. Given the prevalence of gastric cancer and the convenience of follow-up, we believe that using the precise incidence may be a useful index for estimating the occurrence of MMGC.

Among DG procedures, the incidence of MMGC was significantly lower after R-Y than after B-I. There are several plausible explanations for this difference. First, many Japanese patients are thin and the most common reconstruction method after DG is B-I [20]. In contrast, B-II or R-Y are often selected when the extent of the resection is large, the remnant stomach is small, and the duodenal anastomosis is difficult [20]. The remnant stomach’s size would be different between B-I and R-Y, which might explain the difference in the incidence of MMGC. In contrast, the remnant stomach’s size would be similar after B-II and R-Y, although the incidence of MMGC after B-II was significantly higher than that after R-Y. Thus, the difference between B-II and R-Y may be related to the presence or absence of duodenogastric reflux into the remnant stomach, which was experimentally found to promote gastric carcinogenesis [6, 21, 22] and is strongly associated with the development of gastric stump carcinoma [22–24].

Although the standard gastrectomy procedures are DG and TG, the occurrence of post-gastrectomy syndromes remains important [10–13], and PG or PPG are widely used to alleviate post-gastrectomy syndromes. In this context, PG is an alternative to TG for early gastric cancer located in the upper-third of the stomach, while PPG is an alternative to DG for early gastric cancer located in the middle of the stomach. Although these limited surgeries are less likely to cause post-gastrectomy syndromes [25–27], an increased risk of MMGC may be expected based on the large area of the remnant gastric mucosa [6]. The precise incidence of MMGC was 3.01% after PPG, which was statistically equivalent to the incidence after DG, and this result suggests that PPG can be used without concern for the risk of MMGC. However, the risk of MMGC after PG was 2.7-fold higher than that after DG, which agrees with the results reported by Iwata et al. [19]. We also investigated the risks of MMGC after FPG, SG, and LR. The FPG strategy is excellent for minimizing the risk of post-gastrectomy syndromes and ensuring high postoperative quality of life [28], and a prospective nationwide Japanese study is currently evaluating the usefulness of FPG guided by sentinel node biopsy. Assuming that the safety and utility of this procedure are confirmed, FPG would likely become widely used. Nevertheless, the risk of MMGC after FPG is expected to be much higher than that after DG [6]. This study revealed a 3.5-fold higher risk of MMGC after FPG, which was significantly higher than the risk after PG, and this difference indicates that the risk of MMGC increases at larger areas of the remnant gastric mucosa. The difference in residual gastric mucosa may also cause the high incidence of MMGC in PG and FPG than in DG. The remnant gastric mucosa of DG and PPG is mainly the fundic gland, while in FPG and PG, the corpus gland and pyloric gland are widely left behind. Currently, gastric cancers in Japan are still *Helicobacter pylori *(HP)-related cancers. HP-related gastric cancer occurs in atrophic gastric mucosa, and HP-related atrophic gastritis progresses from the pyloric gland to the corpus gland but is poor in the fundic gland.

The development of MMGC can occur not only after gastrectomy but also after endoscopic treatment for early gastric cancer [29]. Abe et al. reported that the cumulative incidences of MMGC after ESD were 9.5%, 13.1%, and 22.7% at 5, 7, and 10 years, respectively [30]. Those results suggest that the incidence of MMGC after FPG seems to be lower than the incidence after ESD. After ESD, HP eradication and endoscopic surveillance are recommended [29–32], and it is important to establish strategies for identifying and managing MMGC after gastrectomy, although these strategies remain unclear. Prolonged endoscopic follow-up is likely necessary, as we found that > 30% of MMGCs were identified at > 5 years, regardless of the surgical procedure. The times from the initial gastrectomy to the MMGC diagnosis were not meaningfully different between the surgical procedures, and the MMGC diagnosis interval was not significantly related to the size and site of the remnant gastric mucosa. However, most MMGCs were diagnosed at > 5 years after PG INT, which is likely related to the difficulty of performing endoscopy after PG INT [33, 34].

As a treatment for MMGC, ESD was more common after PG and FPG (vs. DG B-I), and the incidences of DdMMGC were similar after FPG and DG B-I. This is likely because ESD is easier after PG and FPG (vs. DG), based on the larger size of the remnant stomach and the larger endoscopic working space. If MMGC occurs, ESD may allow the patient to avoid re-gastrectomy, which preserves the remnant stomach and does not impair the patient’s quality of life [35, 36]. The incidence of MMGC after FPG was higher than that after DG; however, ESD could be performed for 68% of FPG cases, which suggests that the choice of FPG would not necessarily be disadvantaged by stomach loss after re-gastrectomy for MMGC [19]. The incidence of DdMMGC was 3% after PG, although this could not be compared to DG B-I and should be interpreted as a 3% disadvantage rate related to the additional TG for MMGC based on the initial choice of PG.

This study has several limitations. First, multiple cancer discovered within 1 year is generally defined as synchronous cancer, rather than MMGC, and this classification is important in terms of carcinogenesis and diagnosis. However, these two diseases have the same clinical treatments and were examined together in this study, which suggests that the true incidence of MMGC might be slightly lower. Second, this study was based on a retrospective questionnaire survey. Third, there was some heterogeneity in the follow-up methods. Fourth, to protect patient privacy and focus on the number of cases, the questionnaire did not collect detailed information regarding patient and family medical histories, HP infection status, use of eradication therapy, the detailed surgical treatments for MMGC, and the patients’ outcomes. A large, multicenter, prospective observational study may be needed to address those issues, although that type of study would be costly, require > 10 years, and encounter difficulty regarding standardization of follow-up methods.

In conclusion, the incidence of MMGC was 2.4% after DG, and there was no significant difference in the incidence after PPG. However, relative to DG, there were significantly higher incidences of MMGC after PG (6.3%) and after FPG (8.2%). This suggests that the incidence of MMGC increased at larger areas of the remnant gastric mucosa. As a treatment for MMGC, ESD was significantly more common after PG and FPG, which suggests that function-preserving surgery can be performed without concern regarding the risk of MMGC. Nevertheless, as > 30% of MMGCs were detected after > 5 years, endoscopic surveillance of the remnant stomach should be performed as long as possible, given the long-term risk of developing MMGC.
